# Development and validation of the modified index of fragility in head and neck cancer surgery

**DOI:** 10.1186/s40463-022-00607-4

**Published:** 2023-01-26

**Authors:** Koorosh Semsar-Kazerooni, Keith Richardson, Véronique-Isabelle Forest, Alex Mlynarek, Michael P. Hier, Nader Sadeghi, Marco. A. Mascarella

**Affiliations:** 1 Faculty of Medicine, McGill University, Montréal, QC Canada; 2 Department of Otolaryngology-Head and Neck Surgery, McGill University Health Center, Montréal, QC Canada; 3 Centre for Clinical Epidemiology, Lady Davis Institute of the Jewish General Hospital, Montréal, QC Canada; 4 Research Institute of the McGill University Health Center, Montreal, QC Canada

**Keywords:** Frailty, Sarcopenia, Cachexia, Head and neck neoplasms, Modified index of fragility

## Abstract

**Background:**

This study aims to develop and validate, a clinically useful modified index of fragility (mIFG) to identify patients at risk of fragility and to predict postoperative adverse events.

**Method:**

An observational study was performed using the American College of Surgeons National Surgical Quality Improvement Program database, from 2006 to 2018. All patients undergoing nonemergency head and neck cancer surgery were included. A seven-item index (mIFG) was developed using variables associated with frailty, cachexia, and sarcopenia, drawn from the literature (weight loss, low body mass index, dyspnea, diabetes, serum albumin, hematocrit, and creatinine). Multivariable logistic regression was used to model the association between mIFG, postoperative adverse events and death. A validation cohort was then used to ascertain the diagnostic accuracy of the mIFG.

**Results:**

A total of 23,438 cases were included (16,407 in the derivation group and 7031 in the validation group). There was a total of 4273 postoperative major adverse events (AE) and deaths, 1023 postoperative pulmonary complications and 1721 wound complications. Using the derivation cohort, the 7-item mIFG was independently associated with death, major AEs, pulmonary and wound complications, when controlling for significant covariates. The mIFG predicted death and major adverse events using the validation cohort with an accuracy of 0.70 (95% CI: 0.63–0.76) and 0.64 (95% CI: 0.63–0.66), respectively. The mIFG outperformed the modified Frailty index.

**Conclusion:**

The modified index of fragility is a reliable and easily accessible tool to predict risk of postoperative adverse events and death in patients undergoing head and neck cancer surgery.

**Graphical Abstract:**

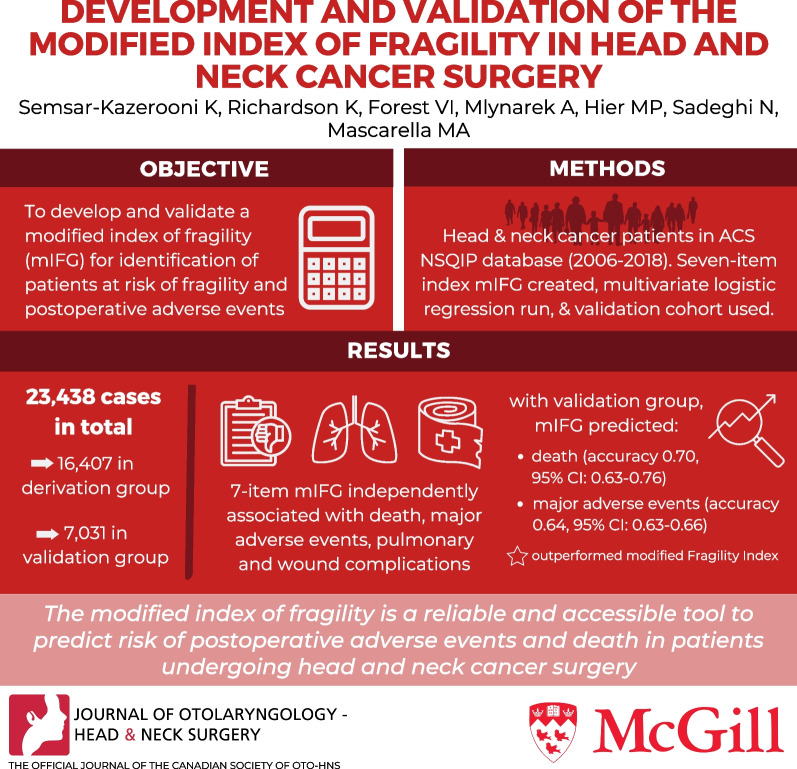

**Supplementary Information:**

The online version contains supplementary material available at 10.1186/s40463-022-00607-4.

## Introduction

Around 40% of head and-neck cancer (HNC) surgeries are associated with postoperative morbidity, which often occurs in the first 30 days after the surgery [1]. While postoperative adverse events (AEs) following surgery have been frequently described in the literature, the factors predicting their occurrence remain poorly defined. Studies in otolaryngology and other oncological surgical fields have shown that cancer cachexia, sarcopenia and frailty are three syndromes that significantly affect patients’ risk of poorer outcomes following surgery [2–4].

Frailty is generally defined as a multisystem dysregulation associated with fatigue, weakness and fat-free weight loss [5]. Sarcopenia can be thought of as a generalized decline in skeletal muscle mass and function [6]. Cancer cachexia, on the other hand, is defined as a metabolic and inflammatory syndrome characterized by ongoing loss of skeletal muscle mass and a progressive functional impairment [3]. These three syndromes have traditionally been studied and conceptualized as different entities, but often co-exist in fragile cancer patients. Indeed, these overlapping conditions share common pathophysiological mechanisms, which present with variable levels of importance in each of these syndromes: imbalance between energy intake and requirements, increased inflammatory activity, muscle wasting, hormonal imbalance and neuromuscular atrophy [7, 8]. These three conditions also share the commonality of being difficult to grade, and thus, to be used clinically, often manifesting as a spectrum of severity, rather than a simple dichotomous diagnosis [9]. All three of these syndromes are highly prevalent in head and neck cancer (HNC) patients, given the anatomical location of most tumors coupled with the invasive nature of treatments, which can often lead to dysphagia [10–13].

Despite differences in their definition, these three overlapping syndromes all lead to body tissue loss and interplay to create a state of fragility in cancer patients. Therefore, the objective of this study is to develop and internally validate an easily accessible pragmatic modified index of fragility (mIFG) to identify patients most at risk of postoperative AEs, including wound complications and pulmonary complications, and death using a large international cohort study.

## Methods

### Study design

A retrospective chart review was performed using the American College of Surgeons National Surgical Quality Improvement Program (ACS NSQIP) database. The ACS NSQIP is an internationally validated, risk-adjusted, outcomes-based program to measure and improve the quality of surgical care. It provides multi-institutional data on postoperative outcomes (up to 30 days) of patients undergoing surgery. In the case of postoperative AEs, the exact time of occurrence is available in the database. Between 2006 and 2018, all patients who underwent nonemergency, inpatient HNC surgery were included in the study using Current Procedural Terminology (CPT) codes. Ethical approval was granted by the McGill University Health Centre research board (MP-37-2018-3568).

### Data collection

Baseline sociodemographic information was noted, including age, gender, ethnicity, alcohol consumption and tobacco usage. Alcohol consumption was quantified as > 2 drinks per day, 2 weeks before the surgical procedure. Active smoking was defined as daily tobacco use within 1 year of the surgery. Comorbidities such as cardiovascular diseases (history of chronic heart failure, myocardial infarction, hypertension on medication, history of chronic obstructive pulmonary disease and dyspnea at rest or moderate exertion), metabolic risk factors (long term steroid usage and diabetes mellitus) and other comorbidities (disseminated cancer, wound infection and preoperative systemic inflammatory response syndrome or shock) were also noted. We included other information such as preoperative laboratory results (levels of creatinine, sodium, hematocrit and albumin), nutritional factors (body mass index (BMI), mean weight and recent loss of body weight in 6 months prior to surgery) and additional operative information (type of surgery, presence of free tissue transfer or tracheostomy and mean operative time) in the data analysis.

### Measurement of outcomes

Postoperative AEs, PPCs, wound complications and death were the outcomes of interest in this study. Pneumonia, unplanned intubation, pulmonary embolism and usage of ventilator for > 48 h were grouped as PPCs. Major postoperative AEs were selected based on the most recent literature and included PPCs, acute kidney injury, cerebrovascular accident, coma, myocardial infarction, cardiac arrest, sepsis, septic shock, more than four blood transfusions, and return to the operating room [14–17]. Wound complications were defined as any of the following: superficial wound infection, deep wound infection or dehiscence. Death, PPC, major AEs and wound complications were coded as binary variables.

### Development of the modified index of fragility (mIFG)

A series of seven preoperative biomarkers and risk factors related to frailty, sarcopenia and cachexia were identified in order to formulate the modified index of fragility (mIFG). The selection of these variables was based on data from the European Working Group on Sarcopenia in Older People (EWGSOP) [18], the international consensus on definition of cachexia by Fearon et al. and by Evans et al. [3, 19], the frailty classification derived by Fried et al. [20]. The following 7 factors were included in the mIFG: loss of > 10% of body weight in 6 months prior to surgery, BMI < 18, chronic baseline dyspnea at rest or moderate effort, blood creatinine levels > 1.35 mg/dL, diabetes, albumin levels < 3.5 g/dL and hematocrit levels < 35% (Table 1).

**Table 1:** Thresholds of the mIFG variables

Variables of the mIFG	Thresholds
Weight loss in 6 months prior to surgery	Loss of > 10% of body weight
BMI	< 18
Chronic baseline dyspnea	0–1
Wound Complications	0–1
Blood creatinine levels	> 1.35 mg/dL
Diabetes	0–1
Albumin levels	< 3.5 g/dL
Hematocrit levels	< 35%

### Weight loss and low BMI

In addition of being an integral part of the definition of cancer cachexia by Fearon et al. and the frailty classification by Fried et al., an unintentional weight loss of > 5% of total body mass, is also associated with sarcopenia and with increased morbidity and mortality rates [3, 19, 21, 22]. In terms of BMI, the frailty classification by Fried et al. also included low BMI as part of their criteria, whereas Fearon et al. consider a BMI < 20 as part of their definition for cachexia [19, 23].

### Surrogate serum markers

Both low hematocrit and low albuminemia [24–26] were reported to be associated with decreased muscle mass and strength in the elderly population. Disturbances in levels of red blood cells could cause limitations of physical function and muscle mass via fatigue and induced local hypoxia in skeletal muscles [27], whereas serum albumin may be an indicator of body protein status, with lower values suggesting a diminished protein reserve, leading to catabolic muscle breakdown [26]. In addition, anemia and albuminemia are both included in the definition of cancer cachexia by Evans et al. [19]. Lastly, there is data confirming high prevalence of sarcopenia in chronic kidney disease, which is also directly associated with anemia [28–30]. It is suggested that high creatinine levels are associated with nearly 80% higher odds of frailty [31].

### Dyspnea and diabetes

As for respiratory effects, sarcopenia has been shown to be associated with shallow breathing and diverse sensory and affective components of exertional dyspnea in patients with chronic respiratory diseases [32, 33]. The generalized muscle weakening often affects respiratory muscles, causing respiratory sarcopenia [34, 35]. As for diabetes, multiple studies have shown high prevalence of sarcopenia and frailty in diabetic patients [36, 37]. Moreover, progressive reduction of muscle mass may increase the risks of insulin resistance and therefore diabetes. Many alterations related to cancer cachexia, including insulin resistance share typical features with type 2 diabetes [38].

The presence of each of the seven aforementioned risk factors was accounted as a score of one, for a minimal mIFG score of zero and a maximal score seven.

### Statistical methods

A total of 43 968 cases were found using the ACS NSQIP database. 23 438 cases met the inclusion criteria of this study using CPT codes for HNC surgery. 70% (16,407) of the cases were randomly assigned in the derivation group and 30% (7031) were randomly assigned in the validation group. Logistic regression was used to identify statistically significant covariates. A multivariate logistic analysis was subsequently performed to study the association between mIFG and major AEs, death, PPC and wound complications. We adjusted for clinically and statistically significant covariates (age, comorbidities, operative time). The validation group was used for interval validity testing. The fit and accuracy of the final model was performed using Hosmer–Lemeshow test. Lastly, the mIFG was compared to existing models such as modified frailty index-5 (mFI-5) [39], American College of Surgeons’ (ACS) risk calculator [40] and American Society of Anesthesiologists (ASA) scores [41] using receiver operator characteristic curves. All tests were performed using R software (R Foundation for Statistical Computing, Vienna, Austria).

## Results

During the 30 days following their surgery, among the 23 438 patients within the derivation cohort, 4273 (18.2%) had a major AEs or death, 1023 (4.4%) had a recorded PPC and 1721 (7.3%) had wound complications. For both the derivation and validation sets, clinicopathological and baseline information, including demographics, social factors, comorbidities, physical measurements and surgical information were collected (Table 2). The distribution of each outcome (Death, PPCs, wound complication and major AEs) is described in Additional file 1: Table S2.

**Table 2:** Comparing baseline characteristics of the derivation group and the validation group

Variables (%)	Derivation cohort (n = 16 407)	Validation cohort (n = 7031)
*Sociodemographic characteristics*		
Gender (male)	10,350 (63.1)	4507 (64.1)
*Age (years)*		
< 50	3451 (21.0)	1524 (21.7)
50–59	3815 (23.3)	1658 (23.6)
60–69	4466 (27.2)	1933 (27.5)
70–79	3049 (18.6)	1272 (18.1)
80–89	1446 (8.8)	564 (8.0)
90+	140 (0.9)	63 (0.9)
*Life habits*		
Current smoker	4036 (24.6)	1825 (26.0)
Current alcohol user	271 (1.7)	109 (1.6)
*Cardiorespiratory comorbidity*		
Exacerbation of CHF^a^	93 (0.6)	38 (0.5)
Myocardial infarct	15 (0.1)	3 (0.04)
Medicated hypertension	7557 (46.1)	3190 (45.4)
Dyspnea	1235 (7.5)	535 (7.6)
Severe COPD^b^	1023 (6.2)	416 (5.9)
*Renal and hematological comorbidity*		
Mean creatinine (mg/dL)	0.95	0.95
Mean sodium (mmol/L)	139.0	139.0
Mean hematocrit (%)	40.0	40.0
*Metabolic comorbidity*		
Long term steroid use	628 (3.8)	252 (3.6)
Diabetes mellitus	2223 (13.5)	996 (14.2)
*Other comorbidities*		
Disseminated cancer	1098 (6.7)	468 (6.7)
Wound infection	577 (3.5)	248 (3.5)
Sepsis ≤ 48 h before surgery	156 (0.1)	55 (0.8)
*Physical measurements*		
Weight loss^c^	721 (4.4)	304 (4.3)
Mean albumin (g/dL)	4.0	4.0
Mean BMI^d^	27.9	27.8
Underweight (BMI < 18.0)	416 (2.5)	158 (2.2)
Normal (BMI 18.0–25.9)	6711 (40.9)	2847 (40.5)
Overweight (BMI 26.0–29.9)	10,602 (64.6)	4507 (64.1)
Obese Class 1 (BMI 30.0–34.9)	13,371 (81.5)	5667 (80.6)
Obese Class 2 (BMI 35.0–39.9)	13,652 (83.2)	5829 (82.9)
Obese Class 3 (BMI ≥ 40)	903 (5.5)	376 (5.3)
*Surgical characteristics*		
Oral cavity	2107 (12.8)	884 (12.6)
Salivary	2555 (15.6)	1147 (16.3)
Neck dissection	3161 (19.3)	1375 (19.6)
Larynx	1076 (6.6)	473 (6.7)
Pharynx	476 (2.9)	201 (2.9)
Craniofacial	557 (3.4)	228 (3.2)
Integumentary	108 (0.7)	44 (0.6)
Reconstruction	1444 (8.8)	615 (8.7)
Other types of surgery	63 (0.4)	30 (0.4)
Cases of free tissue transfers	3043 (18.5)	1313 (18.7)
Cases of tracheostomy	1659 (10.1)	720 (10.2)
Mean operative time (hour)	5.0	5.0

^a^Chronic heart failure

^b^Chronic obstructive pulmonary disease

^c^**> **10% loss of body weight in 6 months prior to surgery

^d^Body mass indices, calculated as weight in kilograms divided by height in meters square

### Modified index of fragility

**Fig. 1:**
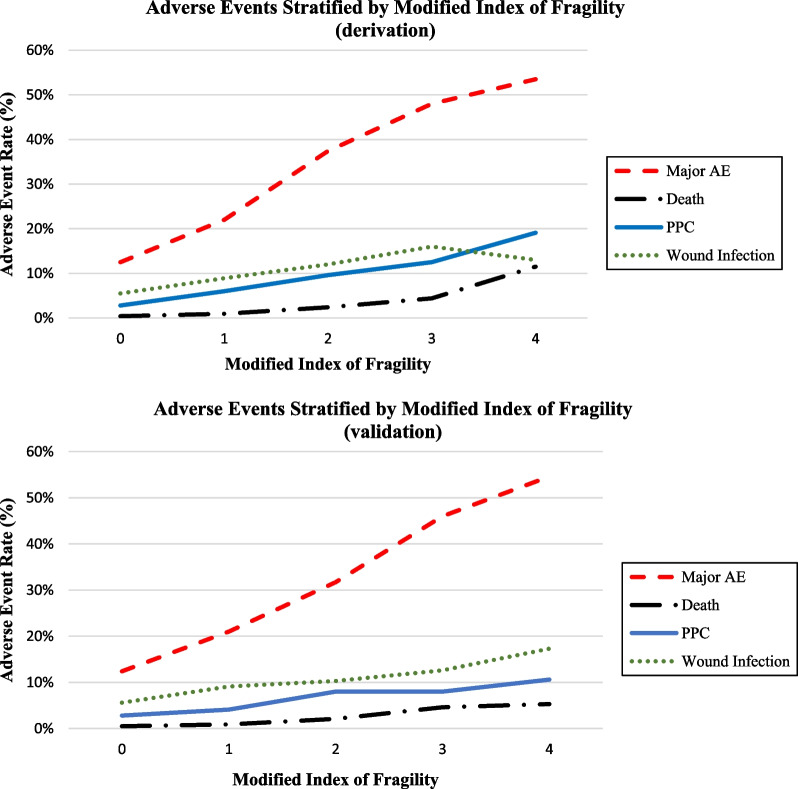
Adverse event rate by mIFG Score. Scores 4 and above are combined

Most patients included in the derivation group had an mIFG score of 0 (10,534, 64.2%). A score of 1 was given to 3872 (23.6%) patients, a score of 2–1365 (8.3%) patients, a score of 3–475 (2.9%) patients, a score of 4–131 (0.8%) patients, a score of 5–27 (0.2%) patients and a score of 6–2 (0.01%) patients. As such, patients with a score of 4 or greater were combined to facilitate statistical analysis. Table 3 demonstrates that mIFG is independent predictor of postoperative wound infections, major AEs, PPCs and death when adjusting for confounding variables. Table 4 and Additional file 1: Table S3 display the comparison of mIFG’s diagnostic accuracy of all four outcomes of this study, in both the derivation and validation group. The mIFG produced a similar area under the curve (AUC) for all outcomes in each of the sub-groups. With increasing score on the mIFG, the rate of AEs increased (Fig. 1).

**Table 3:** Multivariate logistic analysis of predictors of postoperative pulmonary complications, wound complications, major adverse events and death, in the derivation group, when controlling for covariates

	Postoperative pulmonary complications	Wound complications	Major adverse events & death	Death
	OR (95% CI)	OR (95% CI)	OR (95% CI)	OR (95% CI)
mIFG score	1.48 (1.37–1.59)	1.32 (1.24–1.41)	1.75 (1.67–1.84)	2.05 (1.79–2.35)
Sex (male)	1.18 (1.00–1.40)	0.94 (0.82–1.07)	1.06 (0.96–1.17)	1.47 (0.99–2.17)
Tracheostomy	1.59 (1.29–1.94)	1.25 (1.05–1.47)	1.58 (1.39–1.80)	1.37 (0.89–1.85)
Free tissue transfer	1.29 (1.06–1.57)	1.52 (1.30–1.78)	1.94 (1.72–2.18)	1.28 (0.84–1.97)
Current smoker	1.08 (0.90–1.28)	1.45 (1.27–1.66)	1.18 (1.06–1.31)	0.78 (0.52–1.19)
Hypertension (medicated)	1.37 (1.16–1.62)	1.32 (0.71–1.93)	1.07 (0.97–1.18)	1.12 (0.84–1.40)
Severe COPD^a^	1.94 (1.54–2.44)	1.16 (0.93–1.45)	1.34 (1.14–1.58)	1.82 (1.15–2.87)
Operation time (hour)	1.01 (1.00–1.02)	1.01 (1.00–1.02)	1.01 (1.01–1.02)	1.00 (1.00–1.01)
Age (each year of age ≥ 50)	1.02 (1.01–1.03)	1.00 (1.00–1.01)	1.01 (1.00–1.01)	1.03 (1.01–1.05)

^a^Chronic obstructive pulmonary disease

**Table 4:** Comparing mIFG for major adverse events and mortality alone in the derivation and validation group

	Derivation set				Validation set			
	Major AE^a^		Death		Major AE^a^		Death	
	OR (95% CI)	AUC (95% CI)	OR (95% CI)	AUC (95% CI)	OR (95% CI)	AUC (95% CI)	OR (95% CI)	AUC (95% CI)
mIFG score 1	1.99 (1.81–2.19)	0.64 (0.63–0.65)	2.54 (1.64–3.94)	0.73 (0.69–0.77)	1.93 (1.67–2.23)	0.64 (0.63–0.66)	1.69 (0.89–3.24)	0.70 (0.63–0.76)
mIFG score 2	4.13 (3.64–4.67)		5.81 (3.61–9.24)		3.29 (2.74–3.95)		4.15 (2.14–8.04)	
mIFG score 3	6.69 (5.54–8.08)		11.56 (6.67–19.44)		6.02 (4.53–8.00)		9.41 (4.40–20.1)	
mIFG score 4+	7.11 (5.02–10.08)		27.52 (13.88–51.26)		8.80 (5.1–15.13)		10.8 (3.61–32)	

^a^Adverse event

### Area under the curve (AUC)

The AUCs showed that the mIFG had a better ability to predict death among all other outcomes, with an AUC of 0.73 (95% CI: 0.69–0.77) for death, 0.65 (95% CI: 0.63–0.67) for PPCs, 0.64 (95% CI 0.63–0.65) for major AEs including death and 0.60 (95% CI: 0.58–0.61) for wound complications (Table 4). In terms of mortality analysis, the mIFG has the second largest AUC (0.73; 95% CI: 0.69–0.77), right after the ACS risk calculator (0.84; 95% CI: 0.81–0.87). When compared to the mFI-5, the mIFG had a better diagnostic accuracy for the remaining outcomes (major AEs, PPCs, wound complications). As for the ASA Classification, the mIFG was superior in predicting all outcomes except for PPCs: (0.65; 95% CI: 0.63–0.67) versus (0.66; 95% CI: 0.64–0.68) (Table 5).

**Table 5:** Comparing the diagnostic accuracy of mIFG with different risk stratification models

	Modified index of fragility	Modified frailty index-5	ASA^b^ classification	ACS^c^ risk calculator
*AUC (95% CI)*				
Death	0.73 (0.69–0.77)	0.66 (0.62–0.70)	0.69 (0.65–0.72)	0.84 (0.81–0.87)
Major adverse event	0.64 (0.63–0.65)	0.57 (0.56–0.58)	0.63 (0.62–0.65)	0.76 (0.75–0.77)
PPC^a^	0.65 (0.63–0.67)	0.63 (0.61–0.65)	0.66 (0.64–0.68)	
Wound complications	0.60 (0.58–0.61)	0.54 (0.52–0.56)	0.58 (0.56–0.59)	

^a^Postoperative pulmonary complication

^b^American Society of Anesthesiology

^c^American College of Surgeons

## Discussion

The aim of this paper was to develop and validate an easy-to-use index to help identify HNC patients at risk of postoperative complications due to preoperative risk factors related to loss of body tissue. Factors related to frailty, sarcopenia and cachexia (including weight loss, low body mass index, dyspnea, diabetes, serum albumin, hematocrit, and creatinine) were shown to be associated with increased risk of PPCs, wound complications, major AEs and death following HNC surgery. The mIFG showed a satisfactory diagnostic accuracy when compared to other risk-stratification tools, such as the mFI-5, ACS risk calculator and the ASA score.

Risk stratification tools have a major role in today’s surgical practice. A very commonly used example is the ASA scale. The ASA scale is used to subjectively estimate preoperative health status, predict perioperative risk and some postoperative AEs including mortality and cardiac complications [42]. It is formed by general patient characteristics such as presence and severity of systemic disease [41]. Unfortunately, there are currently controversies regarding its use in clinical settings. Sankar et al. reported that the ASA scale has moderate interrater reliability in clinical practice [42]. In this study, the mIFG outperformed the ASA classification in predicting major AEs, death and wound complications. Another commonly used risk assessment tool is the mFI-5 [39]. The mFI-5 uses an accumulation of comorbidities approach to classification and it has been successfully applied to outcomes prognostication in various surgical fields [43–45]. It is comprised by 5 cardiorespiratory and metabolic comorbidities. This score has been shown effective to predict surgical outcomes to certain extent in other surgical specialties [46, 47]. However, given the general nature of its components, its diagnostic ability remains limited. Once again, mIFG had better predicting values for all four outcomes, when compared to mFI. Lastly, the ACS calculator is another decision-support tool comprised of over 20 preoperative factors [40]. It was able to paint a slightly more accurate picture of postoperative death. This was expected given that the ACS calculator is a web-based tool that includes considerably more variables than the mIFG. However, the mIFG is significantly more accessible and easier to calculate in clinical settings.

Frailty, sarcopenia and cachexia remain as well-established negative predictors of surgical and oncologic outcomes. Numerous studies in different surgical fields have reported the association of these syndromes with both long- and short-term postoperative mortality, thus reinforcing, the importance of early diagnosis and management of sarcopenia in surgical candidates [48–50]. Unfortunately, it is often difficult for both researchers and clinicians to have a clear-cut diagnostic tool to determine the presence of frailty, sarcopenia or cachexia [51]. This could be explained by the significant overlap between these 4 syndromes. It could also be due to their multifactorial nature and to the fact that they present as a spectrum of disease severity, rather than a dichotomous diagnosis. In a study on frailty in geriatrics patients, Rockwood et al. developed an index suggesting that frailty, is a result of a set of risk factors accumulated with age, and that this age-related deficit is proportionate to the extent of poor clinical outcomes [52–54]. Similar to frailty syndrome, sarcopenia is multifactorial, affects patients to variable extents, and is associated with poorer outcomes [54]. Cancer cachexia is also classified as a spectrum, ranging from pre-cachexia to severe cachexia syndrome [9, 38]. There are a few validated methods to identify and grade these conditions. For instance, the CACHEXIA score (CASCO), is a score designed to assess for presence of cancer cachexia. However this score is composed of numerous variables including immunological panels that are often not used or accessible in HNC management [55]. Frailty can be assessed through a wide range of frailty instruments [56], each using a variable number of more (e.g., BMI, activity level) -or-less (e.g. memory impairment, mood disorder) available biomarkers and clinical variables. There is however no consensus over which instrument should be favored in the clinical setting, or even on how frailty should really be measured [57]. Sarcopenia can be assessed through radiological indicators such as measurements of skeletal muscle cross-sectional area at the level of the third lumbar (L3) or third cervical (C3) vertebra on computed tomography (CT) scans or dual-energy X-ray absorptiometry, which are not routinely available in the management of HNC [58, 59]. Other measures such as gait speed and grip strength can also be used to assess for the presence of sarcopenia and frailty [5, 18]. However, these tests can often be time-consuming and require trained personnel for accurate and reliable measurements. Most importantly, their efficacy tends to be limited in HNC clinical settings.

Despite the burden of frailty, cachexia and sarcopenia, and clinical barriers to accurately identify patients at risk, evidence indicates that proper intervention, may have a positive impact on prognoses and outcomes of patients undergoing head and neck surgery [60]. Multiple studies have shown the promise of specific interventions in decreasing the heavy burden of these syndromes, such as hormone modulating agents, nutritional supplementation, exercise regimens and appetite simulants [49, 61, 62]. This study highlights the importance of addressing patient’s fragility related to body tissue changes in the preoperative settings, especially in HNC patients who are often at increased risk of dysphagia and malnutrition [63–65]. To our knowledge, this study is the first to describe an easily accessible and pragmatic way to clinically identify patients at risk of postoperative complications due to fragility syndromes (i.e., sarcopenia, cancer cachexia and frailty) in the HNC patient population.

Several limitations of this study could prevent, to a certain extent, the generalization of its results. The variable used to predict risk of fragility are mostly theoretical and drawn from the literature, based on expert consensus. Given that the ACS NSQIP database is limited to postoperative day 30, this study was only able to assess short-term outcomes. This could limit our interpretations of the true extent of impact of these syndromes on postoperative outcomes, especially mortality. Furthermore, the ACS NSQIP database do not address postoperative complications that are specific to HNC surgeries. Specific oncological characteristics, such as staging and type of malignancy, were also not included in the database and we were therefore not able to control for them in our analysis. Some other limitations are inherent to the use of the NSQIP database. Although this database provides a very large sample size and precise data collected rigorously, it does not represent the entire breadth of US hospitals. Previous studies have shown that hospitals participating in NSQIP have differences in patient volume, practice style, and case mix compared with hospitals not in NSQIP [66]. Lastly, in the calculation of the mIFG, each of the seven included variables have the same value; this was intentional to simplify the scoring system. Ideally, determining the true weight of each variable by statistical methods such as beta coefficient calculation would offer the best representation, but may also overfit the data. In addition, this method of calculation favors simplicity and easy access in clinical settings, which was one of the main purposes of developing the mIFG.

## Conclusion

In this study, factors related to frailty, sarcopenia and cancer cachexia were associated with postoperative death, major AEs, wound complications and PPCs in patients undergoing HNC surgery. We also developed and internally validated an easily accessible and simple modified index of fragility that was shown to be a satisfactory predictor of the aforementioned outcomes, when compared to other pre-existing preoperative risk stratification tools. This tool could be a useful complement to pre-operative evaluation and other pre-existing tools. Although there are elements of the mIFG that may possibly be modified pre-operatively (weight loss, low BMI, low hematocrit) further investigation is required to clarify how effective this may be in practice. Nonetheless, implementation of preventive interventions to reduce preoperative sarcopenia in at-risk surgical candidates should have a more significant role in preoperative management of cancer patients. This is particularly important in the HNC population given the high incidence of feeding difficulties before, during and after treatment.

## Supplementary Information

**Additional file 1.** contains further data on comparing mIFG for wound complications and postoperative pulmonary complications in the derivation and validation group in addition to the distribution of PPCs, major AEs, death and wound complication among the cohort.

## Data Availability

All data generated or analysed during this study were provided by the NSQIP and are available upon request.

## Abbreviations

mIFG: Modified index of fragility
AE: Major adverse events
HNC: Head and-neck cancer
ACS NSQIP: American College of Surgeons National Surgical Quality Improvement Program
CPT: Current Procedural Terminology
BMI: Body mass index
ASA: American Society of Anesthesiologists
PPC: Postoperative pulmonary complication
