# Supplementary material for: Development and validation of the modified index of fragility in head and neck cancer surgery
Source: J Otolaryngol Head Neck Surg. 2023 Jan 26;52:5. doi: 10.1186/s40463-022-00607-4 (PMC9878788; doi:10.1186/s40463-022-00607-4)
Supplement: Supplementary file 1 — Additional file 1. contains further data on comparing mIFG for wound complications and postoperative pulmonary complications in the derivation and validation group in addition to the distribution of PPCs, major AEs, death and wound complication among the cohort. [file 40463_2022_607_MOESM1_ESM.docx]

**SUPPLEMENTAL MATERIAL**

| **Supplemental Table 1.** Distribution of PPCs, Major AEs, Death and Wound Complication in Derivation and Validation Group | | |
| --- | --- | --- |
|  | **Derivation Group (n=16 407)** | **Validation Group (n= 7031)** |
| **Major Adverse Events & Death** | 2997 | 1276 |
| **Death** | 150 | 67 |
| **PPCs^1^** | 748 | 275 |
| **Wound Complication** | 1202 | 519 |
| **Major Adverse Events** |  |  |
| Acute Myocardial Infarction | 101 | 30 |
| Acute Renal Failure | 14 | 7 |
| Sepsis | 250 | 97 |
| Stroke | 53 | 31 |
| Bleeding Requiring Transfusion | 1397 | 590 |
| Return to Operating Room | 1398 | 573 |
| Pulmonary Embolism | 66 | 39 |
| Failure to Wean Off Ventilator | 322 | 124 |
| Reintubation | 229 | 101 |

| **Supplemental Table 2.** Comparing mIFG for Wound Complications and Postoperative Pulmonary Complications in the Derivation and Validation Group | | | | | | | | |
| --- | --- | --- | --- | --- | --- | --- | --- | --- |
|  | **Derivation Set** | | | | **Validation Set** | | | |
|  | **Wound Complication** | | **Postoperative Pulmonary Complication** | | **Wound Complication** | | **Postoperative Pulmonary Complication** | |
|  | **OR (95% CI)** | **AUC**  **(95% CI)** | **OR (95% CI)** | **AUC**  **(95% CI)** | **OR (95% CI)** | **AUC**  **(95% CI)** | **OR (95% CI)** | **AUC**  **(95% CI)** |
| **mIFG score 1** | 1.67  (1.45-1.91) | 0.60  (0.58-0.61) | 2.26  (1.89 – 2.69) | 0.65  (0.63-0.67) | 1.67  (1.36-2.05) | 0.59  (0.57-0.62) | 1.51  (1.12-2.02) | 0.61  (0.57-0.64) |
| **mIFG score 2** | 2.33  (1.93-2.80) |  | 3.79  (3.05 – 4.68) |  | 1.91  (1.46-2.52) |  | 3.03  (2.19-4.20) |  |
| **mIFG score 3** | 3.71  (2.90-4.75) |  | 4.88  (3.59 – 6.53) |  | 2.41  (1.58-3.68) |  | 2.62  (1.50-4.55) |  |
| **mIFG score 4+** | 2.34  (1.43-3.80) |  | 6.70  (4.03 – 10.61) |  | 3.48  (1.89-6.42) |  | 4.12  (1.93-8.77) |  |
